# From Displacement to Recovery: A Case Report on Surgical Root Retrieval in the Maxillary Sinus

**DOI:** 10.1155/crid/7967444

**Published:** 2026-04-07

**Authors:** Jacqueline Portelli, Faisal Alzahrani

**Affiliations:** ^1^ Department of Oral Surgery, College of Medicine and Dentistry, Ulster University, Birmingham, UK, ulster.ac.uk; ^2^ Dental Surgeon, Private Practice, Cambridge, England, UK; ^3^ Oral and Maxillofacial Department, Armed Forces Hospital, Southern Region, Khamis Mushait, Saudi Arabia, afhsr.med.sa

**Keywords:** case report, conservative buccal retrieval, maxillary sinus, root displacement

## Abstract

**Introduction:**

Apical displacement of fractured tooth root fragments into the maxillary sinus is a recognised complication during exodontia, particularly in general dental practice. If not appropriately managed, it may lead to adverse sequelae, with heightened risk in medically compromised patients. Although various surgical techniques are described in the literature, management remains case‑specific and sometimes contentious, with no clear guidelines for the dental practitioner. The case presented here is distinctive because it involves a medically compromised patient in whom treatment planning and surgical decision‑making required additional caution to ensure safe, minimally invasive retrieval and successful postoperative recovery.

**Case Presentation:**

A 68‐year‐old medically compromised patient presented with an apically displaced fractured root during extraction of the maxillary left first molar (UL6). Imaging showed that the distobuccal root fragment was entrapped within the Schneiderian membrane of the left maxillary sinus. Surgical intervention for root retrieval was delayed for 2 months due to the patient’s concurrent health issues, during which time the patient experienced persistent facial pain. The root fragment was ultimately successfully removed via a conservative buccal alveolar bone window under local anaesthesia, with minimal postoperative complications and full resolution of symptoms.

**Conclusion:**

This case report contributes to the limited evidence base of conservative alternative techniques to the Caldwell‐Luc approach for fixed‐type displaced root cases in older, medically compromised patients. It supports surgically trained general dentists to safely manage similar cases under local anaesthesia using equipment that is widely available in primary care.

## 1. Introduction

The incidence of fractured root fragments inadvertently pushed apically sub the maxillary sinus Schneiderian membrane or into the maxillary sinus cavity during exodontia ranges from 0.31% to 3.8% in literature [1, 2]. This is based on case reports and small case series reporting root displacement and not on systematic meta‐analysis of a pooled incidence rate of extraction cohorts. The risk is further elevated in cases of excessive apical force in the surgical removal of first maxillary molars, severe maxillary sinus pneumatization and in cases where the alveolar process and Schneiderian membrane are pathologically compromised. If not properly managed, this complication may lead to dental pain, facial pain, oroantral fistula (OAF), acute or chronic rhinosinusitis, mucocele or sinus cysts, fungal infections such as aspergillosis and rarely septic thrombosis of the cavernous sinus, which can be life‑threatening especially in patients with reduced healing capacity or limited surgical tolerance due to medical comorbidities [1, 2]. In rare cases, root fragments have been reported to be expelled by sneezing or nose blowing, inhaled with potential for pneumonia or bronchiectasis or swallowed after passing into the nasal cavity through the ostium [3–5].

Displaced roots are classified into three types [6]: (1) mobile type—the root has perforated the sinus membrane and is free within the sinus cavity, changing location with head positioning and radiographic imaging; (2) fixed type A—the root is inside the sinus cavity but entrapped within the Schneiderian membrane and (3) fixed type B—the root has not perforated the sinus membrane and is located between the membrane and alveolar bone, visible from the alveolar socket immediately post‑extraction. Among the cases reported in literature, the ‘mobile type’ displacement is the most observed.

Although various retrieval techniques have been described—including the alveolar approach [7], the Caldwell‑Luc procedure [8] and endoscopic methods [9]—there remains limited guidance for general dental practitioners regarding when a displaced root fragment can be safely managed in the dental practice. This case report describes a conservative buccal alveolar approach for the removal of a fixed type B distobuccal root fragment under local anaesthesia using commonly available dental equipment and demonstrates the feasibility of this technique in a medically compromised patient.

## 2. Case Description

A 68‐year‐old lady was referred by her general dentist to the Oral Surgery Department at the College of Medicine and Dentistry, Ulster University, Birmingham, United Kingdom. An UL6 root fragment was displaced into the maxillary sinus following extraction of the tooth 2 months prior. A periapical radiograph was attached with the referral letter. The referring dentist did not specify which of the UL6 roots was displaced.

The patient reported a complex and lengthy extraction with severe pain in and around the socket for at least 2 weeks. She presented with persistent dull pain in and around the extraction site, with accompanying throbbing pain and tenderness in the left cheek and persistent headaches. She reported no epistaxis, no nasal discharge or no fluid communication between the oral and nasal cavities at any stage following the extraction.

The patient lived with her husband and smoked 10 cigarettes a day. She smoked heavily for over 50 years but did not drink alcohol. Her medical history included hearing impairment, angina and triple coronary bypass surgery 10 years earlier, as well as pulmonary disease with previous pleurisy and chronic smokers’ bronchitis. She also had type II diabetes, long‑standing depressive illness and anxiety and no known allergies. She was obese with associated mobility difficulties. On examination, she presented with a flushed face, wheezing, shortness of breath and a chronic cough. Her regular medications included metformin 850 mg, aspirin 75 mg, injectable prednisolone for pleurisy and a salbutamol inhaler (Ventolin).

On extra‐oral examination, the left cheek was tender to palpation but not swollen. The mandibular movement was restricted vertically, with a small oral aperture.

Intra‑oral examination showed incomplete healing of the UL6 socket mucosa. The adjacent teeth were periodontally involved but not mobile, not tender to percussion and responded positively to cold testing. The Valsalva manoeuvre produced no fogging of the mirror, blood, discharge, or bubbles from the socket, suggesting there was no oro‑antral fistula.

**Figure 1:**
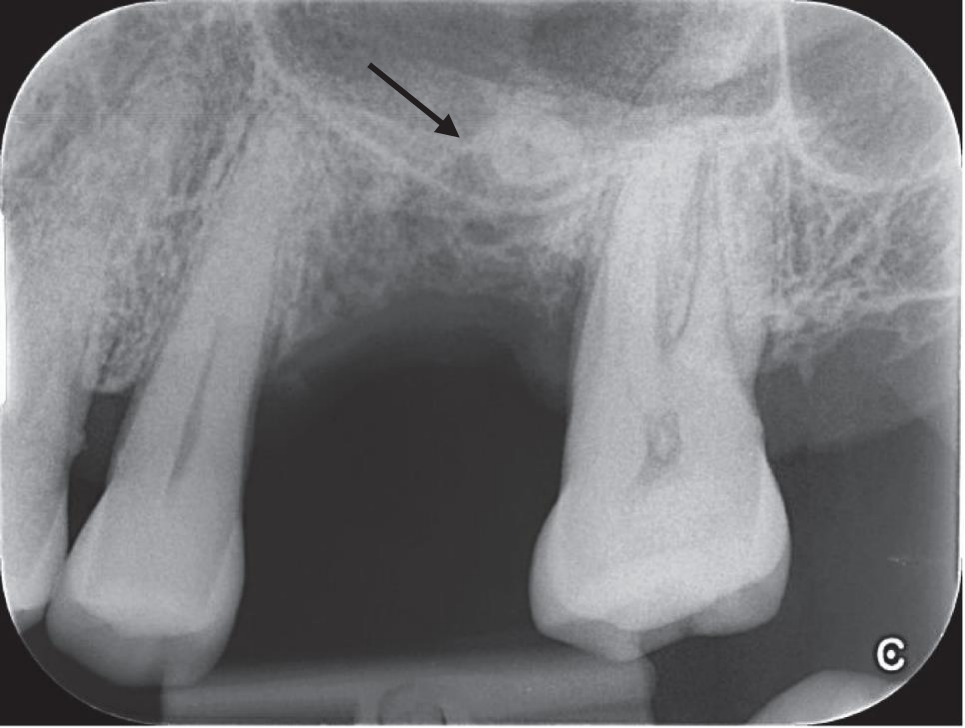
Pre‑operative periapical radiograph showing the distobuccal root fragment in the maxillary sinus region (arrow), which remained in the same position several weeks after displacement.

**Figure 2:**
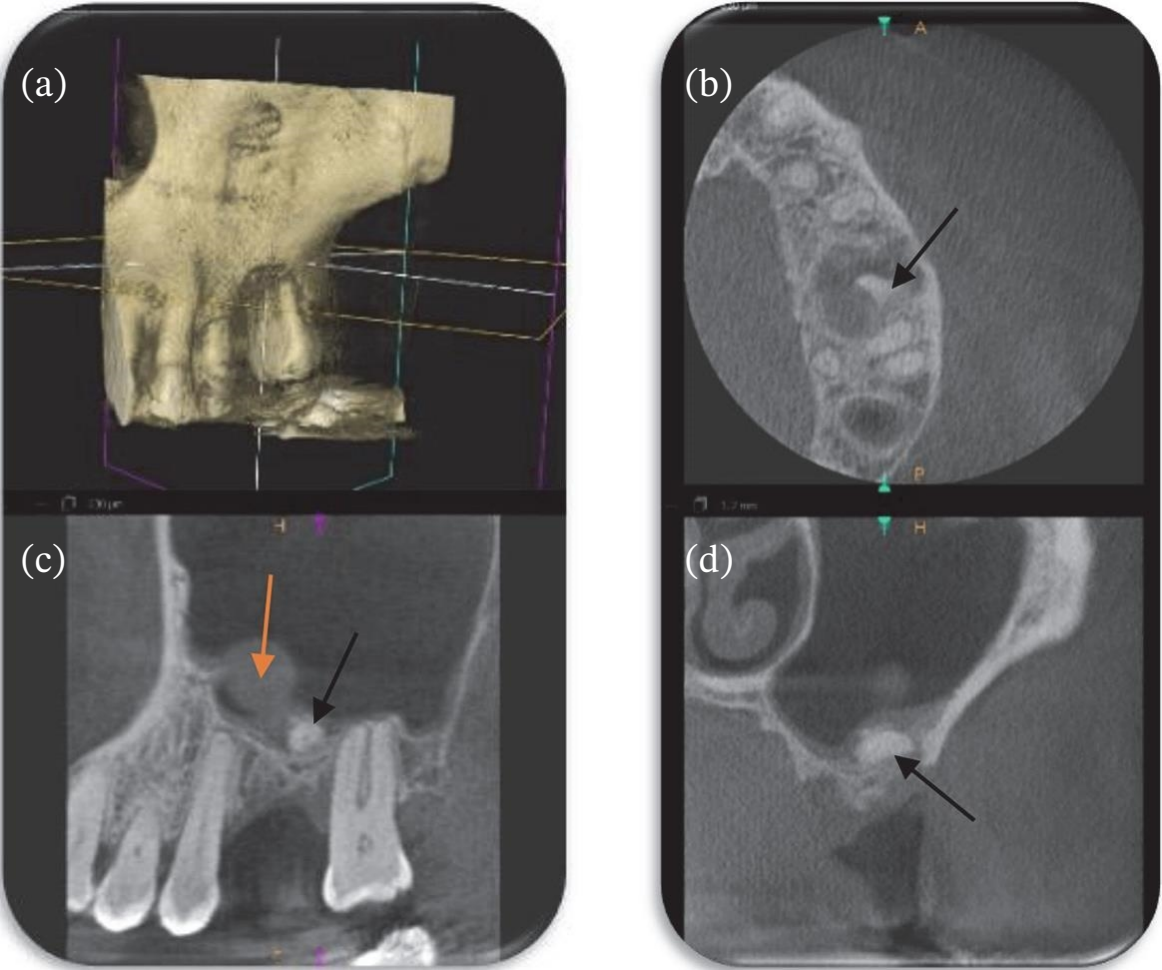
(a–d) The distobuccal root fragment embedded within thickened Schneiderian membrane in the axial, sagittal and coronal CBCT sections (black arrows). A maxillary retention cyst is also visible in the inferior wall of the maxillary sinus (red arrow).

A standard periapical radiograph (Figure 1) and a small field of view (5 cm x5 cm) cone‐beam computed tomography (CBCT) (Figure 2) were taken to locate the root’s exact position. Despite the incident occurring 2 months prior, the radiograph showed no change in the root’s position when compared to the original radiograph sent by the referring dentist. CBCT sections revealed the root to be located beneath the Schneiderian membrane, bucco‐distal of the UL6 socket, with membrane thickening and a small mucosal sinus retention cyst. Treatment options, including monitoring or intra‐oral surgical removal, were discussed. The symptomatic patient preferred and consented to surgical removal. The risks explained included persistent symptoms, severe pain, swelling, infection, bruising, membrane perforation with resultant OAC/OAF, further root displacement, epistaxis and sinusitis.

**Figure 4:**
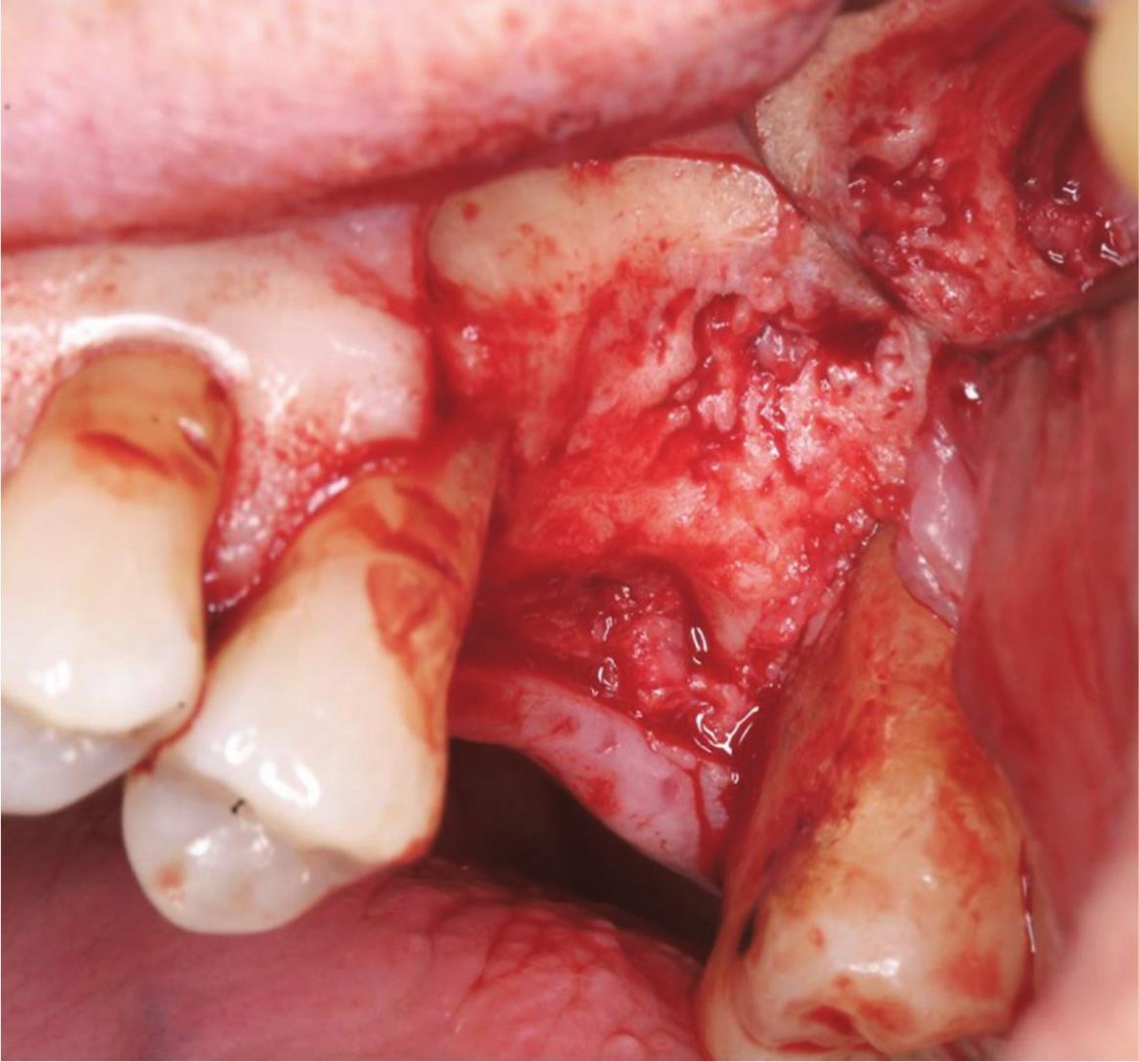
Operative intra‐oral photograph showing a three‑sided full‑thickness mucoperiosteal flap raised to expose the healing alveolus of the UL6.

**Figure 5:**
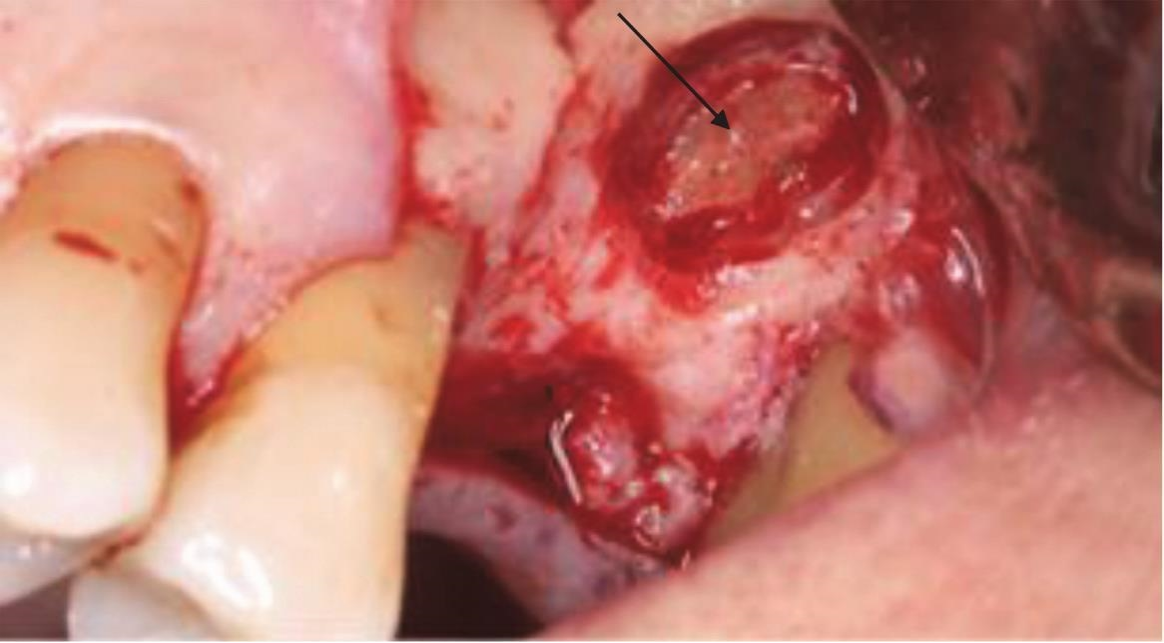
Magnified operative view showing the surgically prepared buccal bone window using a surgical round bur and the retained root fragment (arrow), partially entrapped within the sinus membrane.

**Figure 6:**
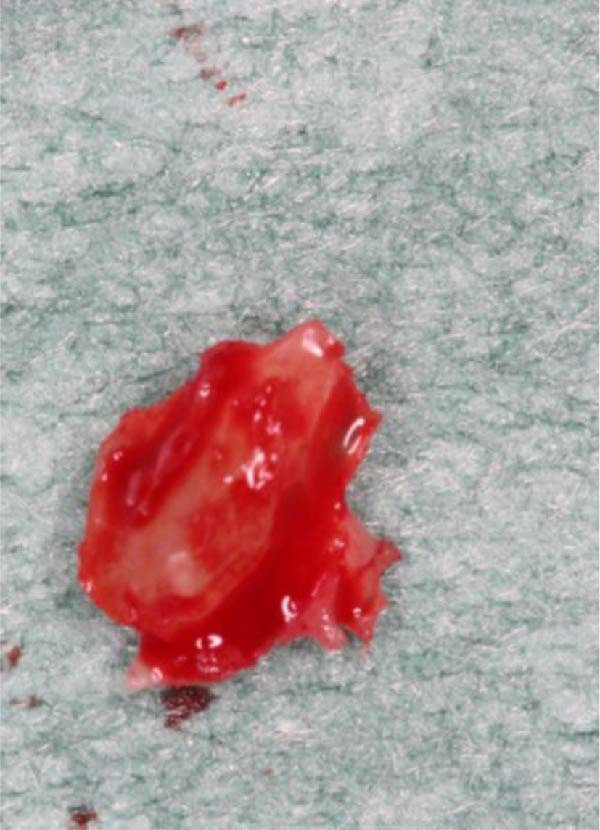
Magnified close‑up view of the retrieved root fragment.

**Figure 7:**
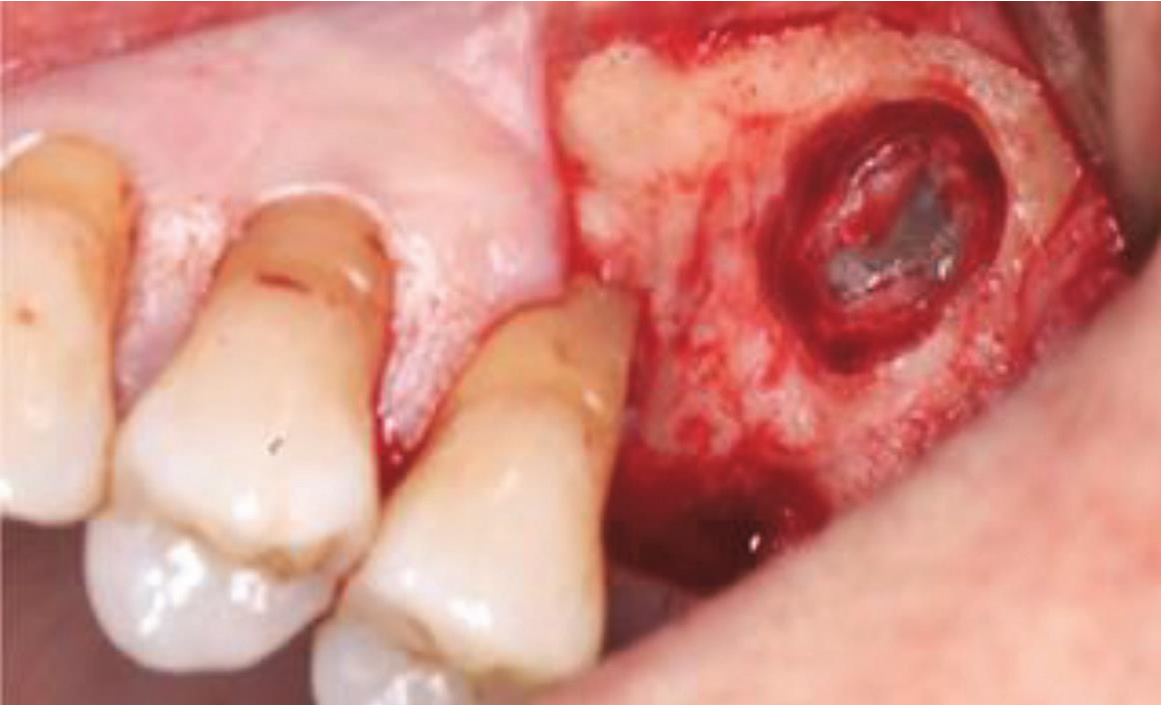
Magnified view of the irregular but non‑perforated Schneiderian membrane following retrieval of the root fragment.

**Figure 8:**
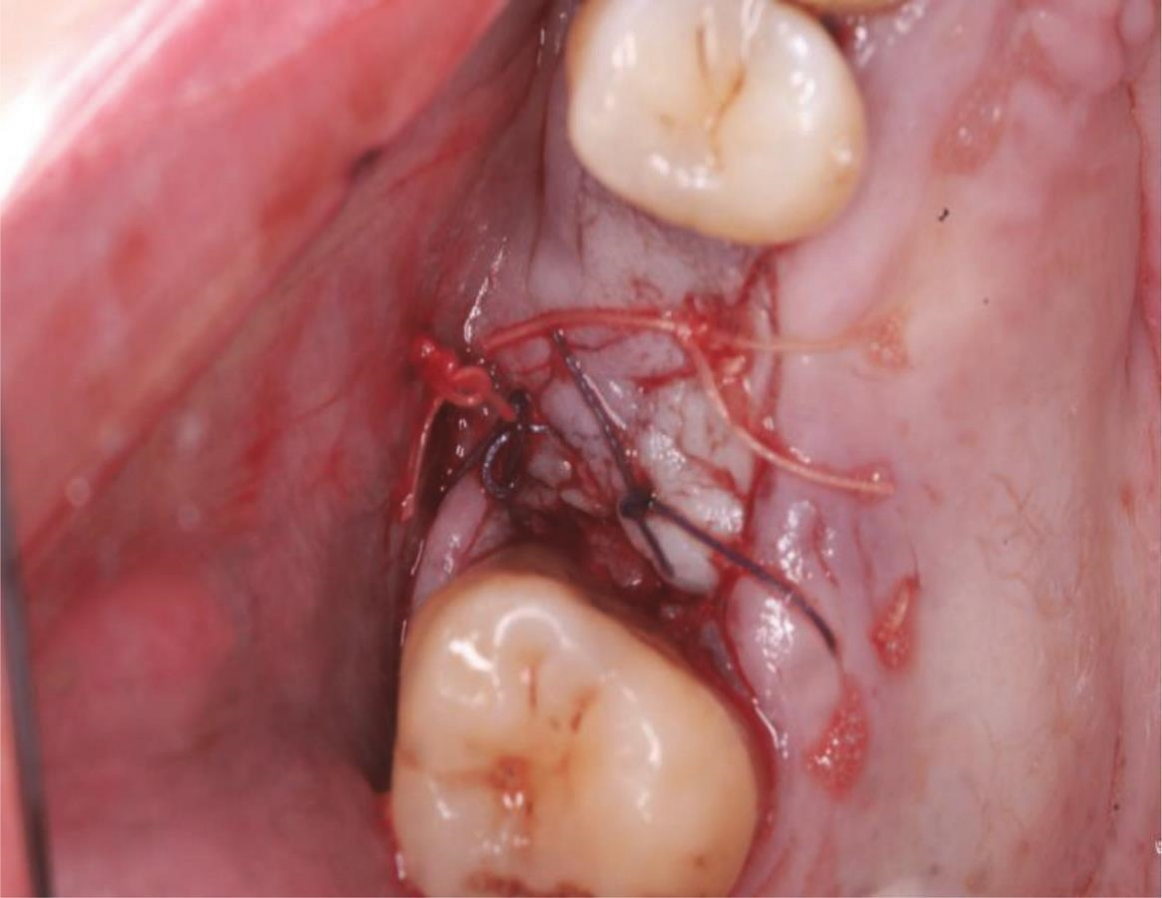
Primary closure of the surgical site achieved using Vicryl 3/0 and 4/0 horizontal mattress and interrupted sutures.

The patient presented for surgery 4 months post‐initial extraction with full mucosal closure but persistent facial discomfort on the left side (Figure 3). The procedure was delayed because of the patient’s need to undergo other urgent medical interventions. Local anaesthesia was administered as 2.2 mL articaine 4% with epinephrin 1:200,000 and 2.2 mL lidocaine 2% with epinephrin 1:100,000 as buccal and palatal infiltrations. A full mucoperiosteal flap consisting of a palatal crestal incision with vertical buccal relieving incisions on the distal of UL5 and the mesial of UL7 was raised using a Molt periosteal elevator (Figure 4). Upon raising the flap, granulation tissue was observed in the buccal alveolar bone of the UL6 socket. A 1 cm x 2 cm bony window was created around this granulation using a straight surgical handpiece with a surgical tungsten carbide round bur (Figure 5). Bone was gently removed over the irregular lateral wall of maxillary sinus, to a depth of ~3 mm. The root fragment was identified and mobilised and retrieved very gently using fine curved tissue forceps (Figures 6 and 7). We were able to extract the root fragment without compromising the integrity of the Schneiderian membrane. After haemostasis was achieved, the flap was sutured with a horizontal mattress and multiple interrupted Vicryl 3/0 sutures for watertight closure (Figure 8). The patient was instructed to refrain from smoking, to withhold blowing her nose for 48 h and to release pressure through her mouth when sneezing to avoid rupturing the sinus membrane. The following medications were prescribed: chlorhexidine rinses 0.12% twice daily and paracetamol 1 g 4–6 hourly pro re nata.

Figure 3(a and b) Intra‐oral views of the surgical site immediately preoperatively, approximately 3 months after root displacement, demonstrating good UL6 socket mucosal closure. No oroantral fistula is evident.

**Figure:**
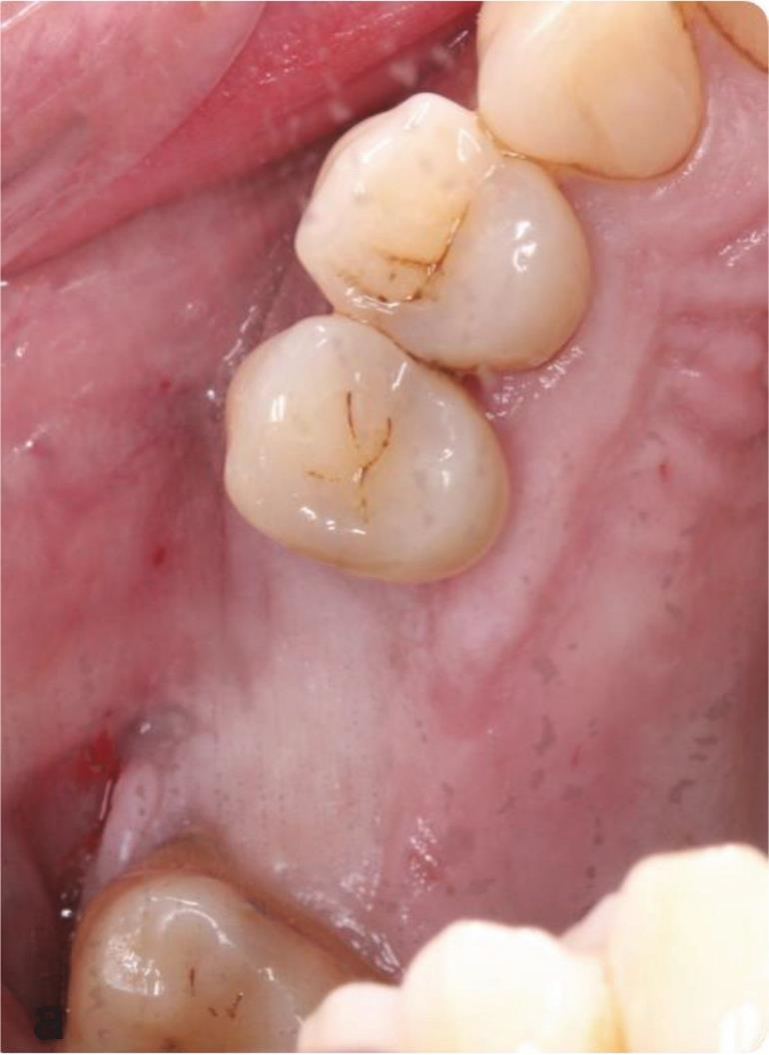
(a)

**Figure:**
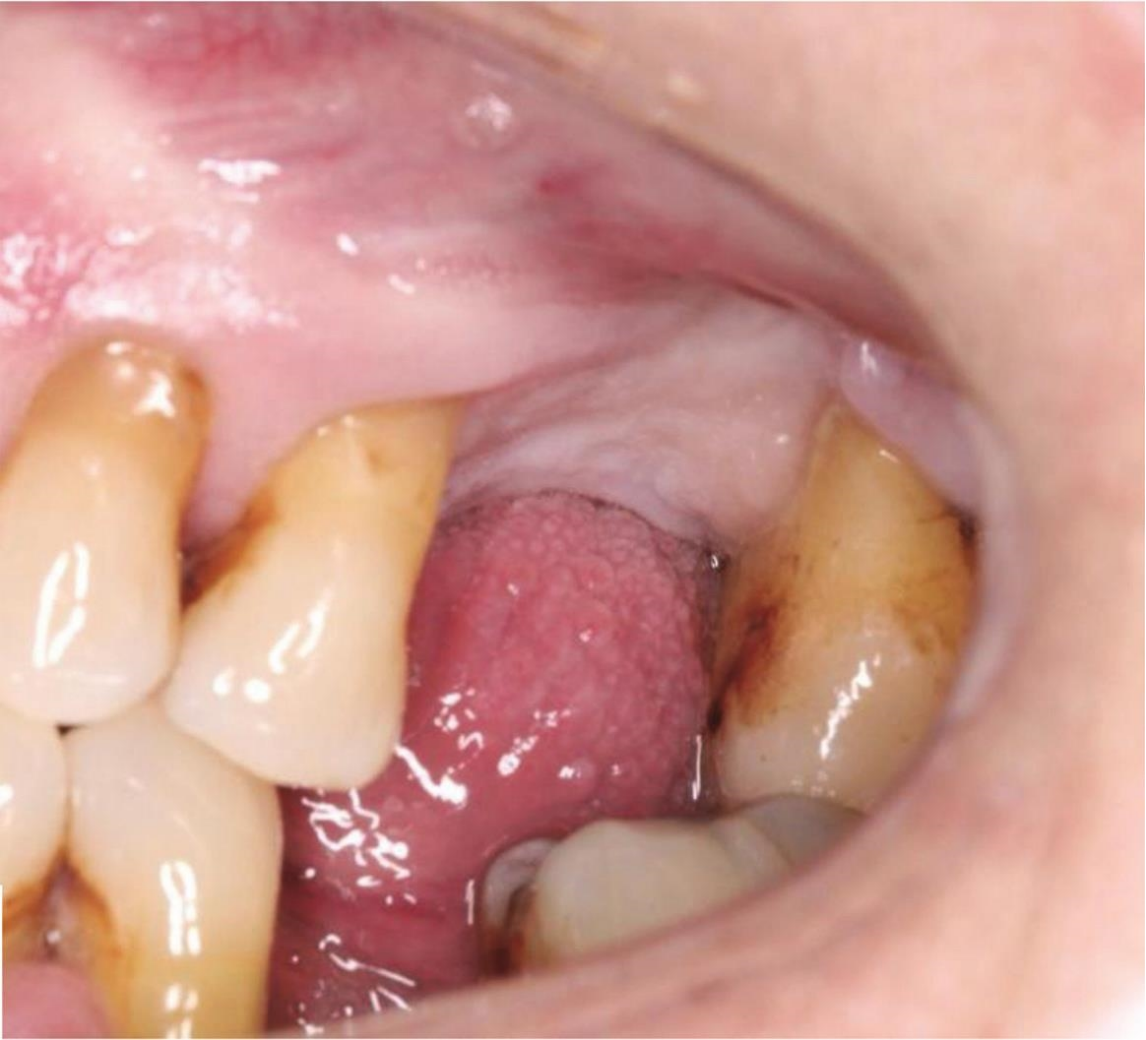
(b)

**Figure 9:**
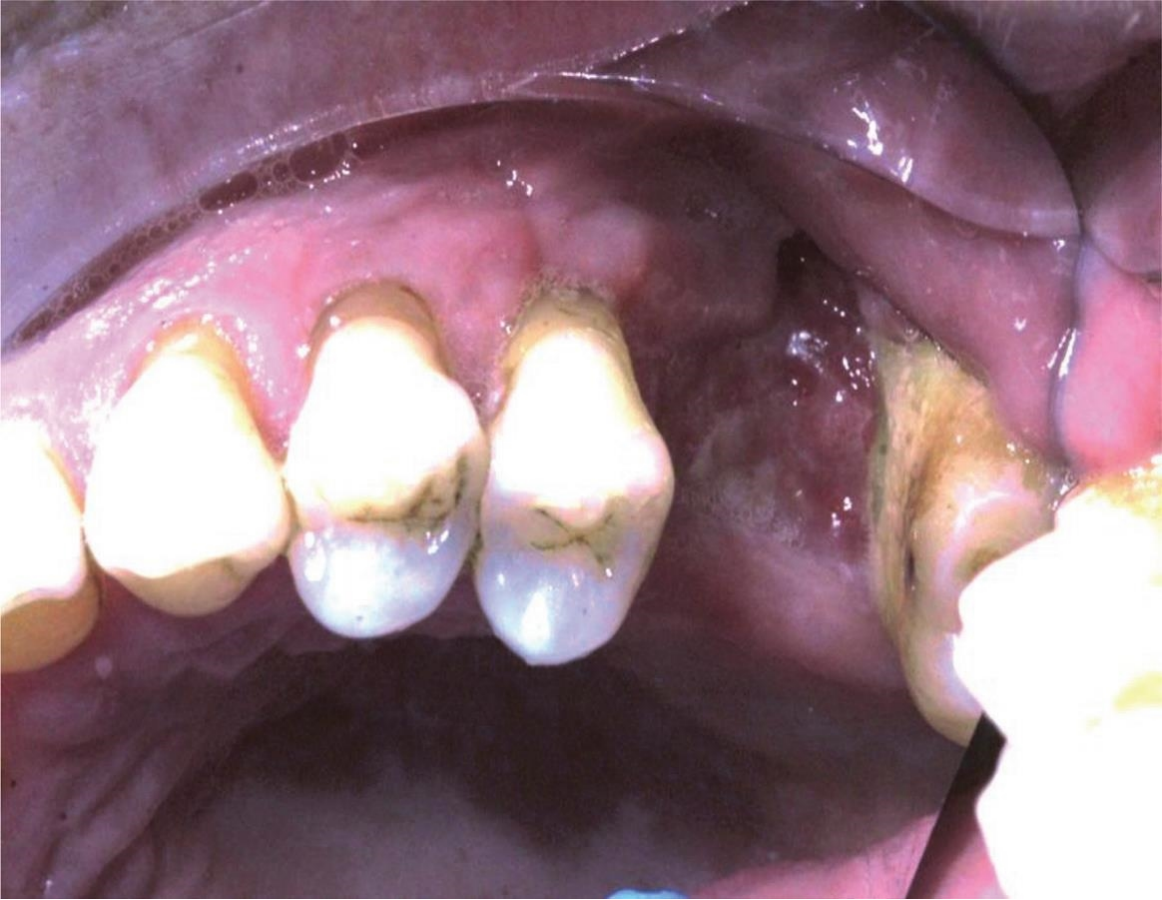
Delayed soft‑tissue healing at the surgical site 8 weeks postoperatively but no oroantral fistula.

**Figure 10:**
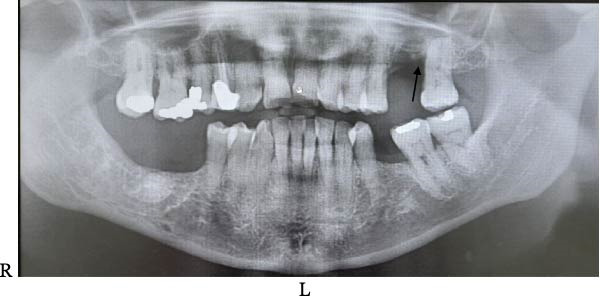
Panoramic radiograph taken 8 weeks postoperatively, showing an intact left maxillary sinus floor (arrow).

The patient reported mild swelling in the cheek area the following day and manageable discomfort. There was no nasal drainage. The socket showed slow healing after 8 weeks (Figure 9). There were no signs or symptoms of OAF. An orthopantomogram was taken to assess the upper left maxillary sinus floor and cavity and to assess a separate ailment in the lower right mandible (Figure 10). It showed no breach in the left maxillary sinus floor. Complete recovery and resolution of symptoms were achieved within 10 weeks. A time‐line summary of events is shown in Table 1.

**Table 1:** Time‐line summary of events.

Interval	Event
Week 1	UL6 extracted in general practice. The distobuccal root was fractured and displaced apically. A referral to the Oral Surgery Department submitted with a periapical radiograph showing the displaced root fragment.
Week 8	The patient attended the Oral Surgery Department complaining of persistent left facial pain. A second periapical radiograph taken. A 5 cm × 5 cm CBCT scan of the UL6 socket obtained. The patient consented for the root retrieval procedure.
Week 16	Surgical procedure delayed because of an urgent medical issue. Surgical retrieval performed.
Week 20	Post operative review (8 weeks post‐surgery) showed delayed healing of the surgical site.
Week 22	Full healing observed (10 weeks post‐surgery). Panoramic radiograph taken.

## 3. Discussion

Removal of the displaced root in the maxillary sinus may cause the general dentist major difficulties regarding its surgical approach and the ability to perform the procedure. There is a scarcity of reliable guidelines on how to approach this complication in the dental office. There is also poor evidence when it comes to the efficiency and complications of the available techniques, with one systematic literature review study calling for more prospective and randomised trials in this regard [10].

Although the current general consensus [1, 3, 11, 12] is that displaced foreign bodies should always be urgently removed from the maxillary sinus to prevent sino‐nasal complications or dangerous spontaneous migration, a small number of authors favour monitoring of foreign bodies in certain cases [4, 7, 13, 14]. They even question whether such a mishap should be regarded as a dental emergency.

Some reports [13, 15] have demonstrated that foreign bodies, including dental implants, can remain in the antrum for several years without or with minimal adverse consequences. However, there are no large studies showing long‐term outcomes of leaving fixed small root fragments under or entrapped within the sinus membrane without removal and no similar case reports involving medically compromised patients.

Selvi et al. [14] reports full healing of an OAC and no sinus complications 7 years after displacement of an amalgam fragment into the sinus cavity post‐tooth extraction, whilst Christmas et al. [13] reports a partially obstructed maxillary ostium with a dental post for several years without any symptoms or pathology. In cases of incidental findings, Selvi et al. [14] suggest monitoring at periodic time intervals if the patient is asymptomatic and there are no findings of inflammation or tissue reactions. Christmas et al. [13], however, stressed that asymptomatic findings are rare, and the authors removed the post regardless, using endoscopy. Manigandan et al. [16] describe the incidental finding of a displaced nasal stud freely mobile inside the maxillary sinus, where the patient remained asymptomatic and no radiographic signs of sinus pathology. It is not reported how long the nasal stud was present inside the sinus.

This controversy and different available approaches can be very confusing for the general practitioner. Both retrieval and retention of a root or foreign body can cause complications, especially in medically compromised patients. Retrieval carries risks such as oroantral communication needing repair, bleeding, infection and damage to adjacent structures.

In this paper, we aim to outline the indications and advantages of a conservative buccal approach compared with other available techniques, with the goal of supporting general dentists in their clinical decision‑making.

Enlargement of the socket with irrigation and retrieval of the fragment using suction or gauze and fibreoptic aids [7, 17, 18] under local anaesthetic is a commonly described non‐invasive approach. However, visibility is poor, and the success rate is reported to be low [18]. This approach can result in loss of maxillary bone for implant placement in the future, risks sinus membrane perforation if the sinus membrane is still intact, with loss of root into the sinus cavity, OAF and sinus infection. In case of the root having perforated the sinus membrane, the dentist may choose to enlarge the existing OAC and approach the removal of the root through this. The advantage of this approach is that it uses the existing OAC and does not create another defect in the antral wall.

When the root is large, mobile, associated with pathology and/or high inside the maxillary sinus, a Caldwell‐Luc approach is generally preferred by oral surgeons because it allows direct visualisation. The visualisation of the root can also be assisted by an endoscope. Apart from foreign‐body removal, it allows sinus clearance and drainage, especially in the presence of serious mucosal disease. The procedure is, however, invasive, with the risk of postoperative sinusitis, altered sensation due to infraorbital nerve damage, facial asymmetry, swelling, dental problems and heavy bleeding from the maxillary artery [18]. The large cavity may also violate the integrity of the sinus wall, although a replaceable lid approach using piezo handpiece has been described in literature to overcome this problem [19].

Nasal endoscopy allows root retrieval using a minimally invasive, reliable, safe procedure that respects the integrity of the sinus, involving no bone or soft tissue cutting. It facilitates recovery and provides excellent visibility [20]. However, minimally invasive access is inadequate for foreign bodies of large dimensions. The procedure needs to be performed under general anaesthesia or sedation. Specific training and equipment which are not available in a dental office are required and the procedure is normally carried out by a rhinologist. If the root fragment is close to the anterior or inferior wall of the maxillary sinus, it is almost impossible to retrieve it with a trans‐nasal approach. Furthermore, the presence of an oro‐antral fistula will need a combined oral surgical approach.

A modified approach to the Caldwell‐Luc procedure is not a novel phenomenon. However, reports specifically describing the retrieval of root fragments via a conservative buccal window remain scarce in the literature, with only a few documented cases [4, 21–23]. Notably, these cases involved relatively young and otherwise healthy patients, which may limit generalizability. The majority of recently published papers focus on the use of this technique for retrieval of displaced dental implants [24–26]. Although implants and root fragments share the risk of further migration once displaced, they differ in key ways. Dental implants are sterile, rigid foreign bodies with predictable geometry, whereas root fragments are biologically derived, variable in form, and may carry pathological tissue. These differences affect both retrieval difficulty and complication risk.

Migrated implants can trigger chronic foreign‑body reactions, mucosal hypertrophy, or sinusitis, but their size and cylindrical shape usually prevent spontaneous passage through the maxillary ostium unless it is pathologically enlarged. Root fragments, however, are smaller, commonly enter the sinus during extraction, and can pass through the ostium into the nasal cavity. Their non‑sterile nature also increases the likelihood of infection [10, 24–27].

It must be emphasised that the conservative buccal window technique described here is not a universal solution for all cases of root displacement into the sinus. It is the authors’ opinion that displacement of a root fragment represents a significant complication that warrants timely and thorough investigation. Where available, CBCT should be employed without delay. Although the position of a root fragment can be located by 2D imaging using parallax techniques, CBCT provides superior visualisation of the fragment’s location, size, orientation and any associated maxillary sinus pathology, thereby guiding the most appropriate surgical approach. Only once the root is located, its size determined and the sinus cavity is evaluated for pathology can the best course of treatment for the patient be instigated.

Management needs to be on a case‐to‐case basis and with the consent of the individual. Our case described a displaced fixed distobuccal root, but when a palatal root is fractured, retrieval can be more complex. Although the conservative buccal approach can be used for displaced palatal root fragments under/entrapped within the sinus membrane, the procedure is more technically demanding under local anaesthesia and not always the safest or most efficient route. The limited visibility caused by thicker palatal bone complicates retrieval. In a case report by Borgonovo et al. [23]. The clinicians intentionally left a displaced palatal root fragment beneath the Schneiderian membrane within the palatal alveolar bone after extraction, planning to retrieve it later through a conservative buccal lateral window while simultaneously placing an implant. This approach was carried out successfully.

The patient in our reported case was obese and diabetic with pulmonary disease. She presented many challenges and problems of management. Besides systemic complications during treatment, her medical complications risked healing problems and infection. Furthermore, the patient had anxiety issues with a small oral aperture. Therefore, the risks of leaving the roots in situ with further future infections and pain had to be balanced against the risk of undergoing an invasive surgical procedure. The options were well discussed with the patient. With a more inferiorly and buccally placed non‐mobile, non‐cystic root, it was possible to access the root conservatively. A widely available surgical bur and motor were used to create the window, and the Schneiderian membrane was not perforated during the procedure. In patients with a history of heavy long‐term smoking, the sinus membrane is often thicker and more resistant to tearing [28], which can make the use of a bur particularly safe and practical. Conversely, in cases where the membrane is thin or fragile such as in young non‐smoker healthy patients, piezoelectric devices are preferred due to their selective cutting and reduced risk of membrane perforation. Thus, the choice of instrument should be tailored to patient‐specific factors, balancing availability, operator experience, and the thickness of the alveolar bone and the sinus membrane.

Major complications such as oro‐antral communication, infraorabital nerval damage, orbital haematoma, visual disturbances, bleeding and rhinosinusitis were avoided in this case, with full resolution of symptoms.

## 4. Clinical Significance and Contribution to Literature

Although our approach is a well‑established technique in oral surgery, no studies address outcomes in elderly or medically complex individuals. Furthermore, no systematic review specifically evaluates root fragments or other foreign bodies displaced into the maxillary sinus managed using a conservative buccal approach. This represents a clear gap in the literature, limiting guidance on case selection and on the safety and applicability of conservative buccal approaches for root displacement in older or comorbid patients.

## 5. Conclusion

This case demonstrates that a displaced fixed molar root fragment beneath or entrapped within the sinus membrane can be safely retrieved through a conservative buccal window under local anaesthesia, even in a medically compromised elderly patient. It shows that surgically trained general dentists using standard equipment can successfully manage similar situations. While this case adds to the growing evidence base, further structured case series and observational studies are needed to better define expected outcomes, refine case selection and develop clearer clinical guidelines for managing comparable cases.

## Author Contributions

**Jacqueline Portelli**: conceptualisation, operator of clinical procedure, writing – original draft, writing – review and editing of subsequent drafts and final version, submission of CARE checklist, project administration. **Faisal Alzahrani**: visualisation (clinical photography and image editing), writing contribution of original draft, review of subsequent drafts.

## Funding

No funding was received for this manuscript.

## Ethics Statement

The authors have nothing to report.

## Consent

Written informed consent has been obtained for the use of images for individuals included in the article.

## Conflicts of Interest

The authors declare no conflicts of interest.

## Data Availability

Data sharing is not applicable to this article, as no datasets were generated or analysed during the current study.
